# Latrine Ownership and Its Determinants in Rural Villages of Tigray, Northern Ethiopia: Community-Based Cross-Sectional Study

**DOI:** 10.1155/2020/2123652

**Published:** 2020-08-17

**Authors:** Kiros Fenta Ajemu, Abraham Aregay Desta, Asfawosen Aregay Berhe, Ataklti Gebretsadik Woldegebriel, Nega Mamo Bezabih

**Affiliations:** Tigray Health Research Institute, Mekelle, Tigray, Ethiopia

## Abstract

**Background:**

Open defecation was largely a rural phenomenon most widely attributed to poor latrine ownership at community level. We aimed at examining latrine ownership and its determinants in rural villages of the Tigray region, Northern Ethiopia.

**Methods:**

Community-based cross-sectional study was conducted from June to July 2018. A total of 756 randomly selected households were involved in the study. The multistage cluster sampling technique was used to select study households. Data were checked, coded, and entered into Epi-Info version 7. Besides, it was exported to SPSS version 20 for data analysis. Multivariable logistic regression analysis was involved to estimate the net effect size of factors associated with latrine ownership.

**Results:**

The proportion of households owning latrine was 35.7%. The majority (84.4%) of constructed latrines were utilized by household families. Households advocated latrine IEC by Health Extension Workers (HEWs) (AOR = 1.902, 95% CI: 1.269–2.852), living in their private house (AOR = 3.13, 95% CI: 1.528–6.401), and the occupation status of government employees (AOR = 3.54, 95% CI: 0.586–21.397) are more likely to lead to the construction of latrines. The availability of latrine made on slab floor (AOR = 1.790, 95% CI: 0.297–3.102), having a latrine constructed inside the household compound (AOR = 4.463, 95% CI: 1.021–19.516), and delivery of latrine IEC by Women Development Armies (WDAs) (AOR = 2.425, 95% CI: 0.728–8.083) may lead to better latrine utilization at the household level.

**Conclusion:**

Households owning latrine at the community level were low. The desired level of latrine ownership will be realized if all sanitation and hygiene components are kept on eye side by side in line with identified predictor factors.

## 1. Introduction

Globally, more than 2.5 billion people lacked improved sanitation and hygienic facilities [1]. A disease associated with poor water, sanitation, and hygiene accounts for more than 4% of the total disease burden and deaths [1–3].

Open defecation was largely a rural phenomenon most widely practiced in the southern part of Asia and Sub-Saharan Africa. This was due to poor sanitation and hygienic practice. In Sub-Saharan Africa, an estimated one-third of the population still do not have access to improved hygiene and sanitation facilities [4–6].

In Ethiopia, only six percent of household members were reported using improved hygiene and sanitation facilities. Some evidence suggested that financial constraints of raw materials needed to construct latrine and poor interest of the household members to construct latrine rented house with occupancy of the landlord were factors for enhancing sanitation and hygiene facilities at the community level [3]. Latrine ownership in the rural community was low, resulting in high burden of diarrheal and other communicable diseases. It is evident from Ethiopian Demographic and Health Surveys (EDHS, 2016) that an estimated 56% of the rural households practiced using unimproved latrine [7]. Meanwhile, urban areas were four times more likely to own latrine at the household level [3, 7]. Pooled latrine prevalence (50.02%) was reviewed from 17 community-based studies in Ethiopia. The highest prevalence (67.4%) was documented in Southern Nations Nationality and People Regional State (SNNPS) followed by Amhara Region (50.1%), almost consistent with the national estimate. Respondents who completed high school and above education levels were more likely (1.79; 95% CI: 1.05–3.05) to own latrine at the community level as compared to their counterparts [8].

In the Tigray region of northern Ethiopia, different fragmented small pocket studies have been conducted to assess the level of latrine ownership at the community level in different areas within the region. However, the reported findings were with highly varying figures. Regarding latrine utilization, more than half (59.8%) was reported from the La'ilay Maichew district of central Tigray and Enderta district of Mekelle Zone (68.4%) that showed a significant variability [9, 10]. Meanwhile, latrine prevalence was only reported from South Eastern part of Tigray (53%) [11]. Community members familiar with community lead total sanitation (CLTS) (1.78; 95% CI: 1.57–2.03), accessibility of health facilities near the rural village (2.37; 95% CI: 2.14–2.64), and increased educational attainment of the head of the household were positively influenced the households to own latrine at community level [9].

**Figure 1:**
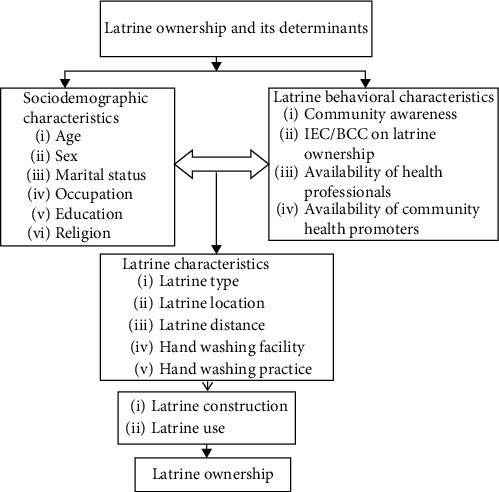
The latrine ownership ladder: a conceptual framework for enhancing sanitation uptake in low-income settings [22].

Increasing latrine prevalence and utilization are essential and a cost-effective strategy to overcome the disease burden associated with improper excreta management by strengthening latrine ownership [12–15]. Latrine ownership is affected by a range of behavioral, cultural, social, geographic, and economic factors across different community members [11, 16–21]. However, reviewed determinant factors of latrine ownership at the regional level in the previous studies were limited to behavioral factors. Besides, none was documented concerning latrine ownership that showed the regional estimate. Therefore, we aimed to use the latrine ownership ladder model [22] (Figure 1) to assess the latrine ownership and its determinant factors in rural villages of the Tigray region, Northern Ethiopia.

## 2. Methods and Materials

### 2.1. Study Design and Settings

**Figure 2:**
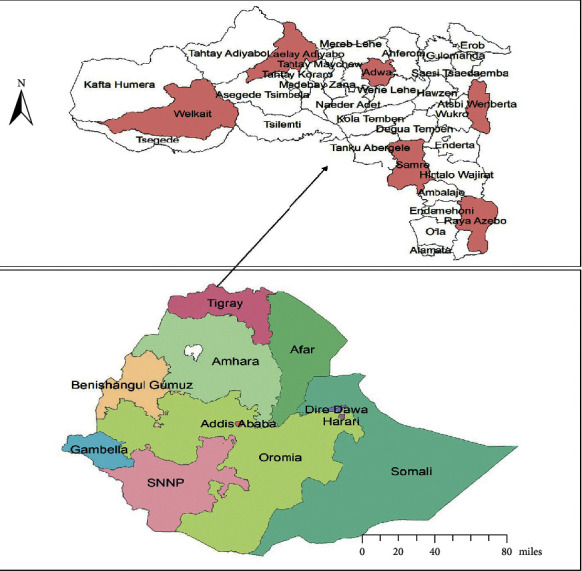
Map of the Tigray region and selected study districts were shaded by rose color.

The study involved community-based cross-sectional survey. It was conducted in rural villages of Tigray, Northern Ethiopia from June to July 2018. Tigray region is among the nine regional states administratively demarcated and it is located in the northern part of Ethiopia at a distance of 805 kms from the capital city, Addis Ababa. The region is further administratively subdivided into seven zones, namely, East, South, South East, Western, Northwestern, Central, and Mekelle which contained the smallest administrative units of 52 districts (34 rural and 18 urban) [23] as shown in Figure 2.

### 2.2. Sampling and Sample Size Determination

**Figure 3:**
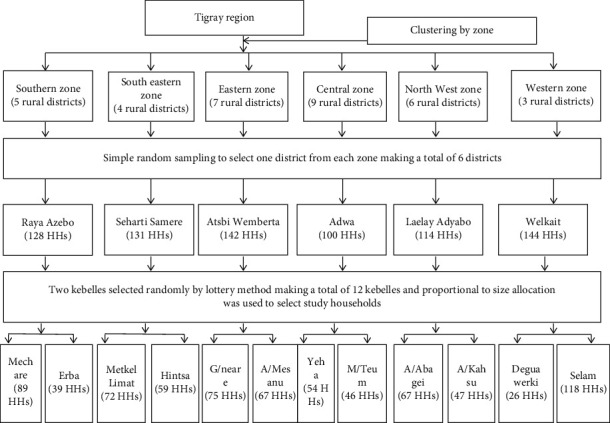
Schematic diagram of the sampling procedure.

Multistage cluster sampling techniques were used to select study households. Administrative zones and districts were used as a cluster. Of the seven clusters, one zone (Mekelle) was excluded since it was urban. From the six rural zones, one district was randomly selected making a total of six districts. Meanwhile, twelve kebelles were randomly selected by the lottery method considering two kebelles from each district. Finally, proportional to size allocation was used to select a total household that participated in the study (Figure 3).

The sample size was determined by using single population proportion formula with an assumption of 34% latrine prevalence (*P*=0.34) from a previous study [24], *Z*-score *Z*_(*α*/2)_ = 1.96 at a confidence level of 95%, and a margin of error *ε* = 0.05 (5%). The sample size was 690. Since the sampling procedure was multistage, we considered the design effect of 2 and 10% nonresponse rate making a total sample size of 759.

### 2.3. Data Collection Procedure

We used a standardized pretested structured questionnaire for data collection adopted from previously conducted similar studies [11, 12]. During the data collection procedure, face to face interviews were conducted by the head of the household. Besides, direct observation was involved with household compounds to confirm the availability of latrine, type of latrine, its location, and how it was utilized. The data collection tool was originally prepared in English and translated to Tigrigna (local language), and responses were then translated back to English. Data collection was conducted by recruiting six trained environmental health professionals. Data collection was conducted under the close supervision of the principal investigators.

### 2.4. Data Quality Assurance

To enhance data quality, training was provided for data collectors and supervisors with the objective of the study, the nature of the data collection tools, ways of approaching during interviews, observation, and inspecting latrines. During the data collection period, there was a strict supervision scheme. Completed questionnaires were checked on a daily basis by a supervisor and principal investigator.

### 2.5. Operational Definitions

- Latrine ownership: the proportion of households owning and utilizing latrine [7].
- Hand washing facility: any setup of a container with water and soap in the household compound for hand washing purposes observed at the time of data collection.
- Women Development Armies: women enrolled under 25–30 teams for implementing health extension package at the community level.

### 2.6. Data Processing and Analysis

Data were entered into Epi-info version 7 and exported to SPSS version 20 statistical package for analysis. Frequency distribution and percentages were performed using frequency tables. Bivariate logistic regression analysis was done for each outcome variable for latrine ownership (latrine prevalence and utilization). All variables at *P*-value of ≤0.25 in the bivariate analysis were included in the multivariable logistic regression model. Finally, all variables with *P*-value of ≤0.05 were considered statistically significant as predictor factors.

## 3. Result

### 3.1. Sociodemographic Characteristics

A total of 756 (99.6%) households completed the study. The majority (98%) of the respondents were Orthodox Christian. More than half (58.2%) were unable to read and write (Table 1).

### 3.2. Latrine Ownership and Its Characteristics

The study revealed that 270 (35.7%) surveyed households owned latrine at the community level. More than half (64%) constructed latrines were made of wood and mud floor and were found inside the household compound. Regarding their location, 90.7% were nearly 50 meters away from the kitchen as per standard. Besides, 84.4% of constructed latrines were utilized by household members. Accordingly, the majority (87%) of adults and children utilized latrine regularly. The majority (85.2%) of constructed latrines lacked hand washing facilities. The very low proportion of family members (5.6%) washed their hands with soap and water after latrine use (Table 2).

### 3.3. Determinant Factors for Latrine Ownership

The study revealed that households advocated latrine IEC by Health Extension Workers (HEWs) (AOR = 1.902, 95% CI: 1.269–2.852), living in a private house (AOR = 3.13, 95% CI: 1.528–6.401), and the occupation status of government employees (AOR = 3.54, 95% CI: 0.586–21.397) were more likely to lead to the construction of latrines. The availability of latrine made on slab floor (AOR = 1.790, 95% CI: 0.297–3.102), having a latrine constructed inside the household compound (AOR = 4.463, 95% CI: 1.021–19.516), and delivery of latrine IEC by Women Development Armies (WDAs) (AOR = 2.425, 95% CI: 0.728–8.083) may lead to better utilized latrine at the household level (Table 3).

## 4. Discussion

This was the first regional estimate to latrine ownership and its determinants among rural residents in the Tigray region, Northern Ethiopia.

The study revealed that households that owned latrine were 35.7%. Five out of six constructed latrines were utilized by household members. Accordingly, latrine prevalence was almost consistent with the national estimate (44%) as documented in EDHS, 2016 [7]. Similar evidence was reported from South East Zone of Tigray, Northern Ethiopia (37.6%), Central Zone of Tigray, Northern Ethiopia (37.4%), and Dabat district, North West Ethiopia (47%) [11, 25, 26].

As to the factors responsible for latrine ownership, households living in their own house were 3.3 times more likely to construct latrines as compared to their counterparts. This was consistent with the finding in Dabat district, North West Ethiopia, in which households in rented houses had a difficulty in constructing latrines [25]. This finding showed the need for extraordinary attention and awareness creation to address this special community groups.

Study finding showed latrine ownership is low when compared to elsewhere in Bahirdar Zuria of Northern Ethiopia (58.4%) [24], Debere Tabor in Amhara region, Northern Ethiopia (93.5%) [12], and Oromya region, South West Ethiopia (88.2%) [27]. Besides, a report from the current study was lower when compared with evidence from Nepal [17]. The discrepancy might be due to the recruitment of graduated model households due to the implementation of Community Led Total Sanitation (CLTS) in Bahirdar and enrollment of urban households in the remaining two studies. In contrast, the present study recruited all household members from the rural community.

Regarding latrine utilization, it was higher than evidence from systematic reviews at the national level (37.7%), Central zone of Tigray, Northern Ethiopia (58.9%), Awabel district of Amhara region (52%), and Gulomekeda of Tigray region (57.3%) [8, 9, 19, 28]. Latrine location inside the house compound was four times more likely utilized by household members (AOR = 4.463, 95% CI: 1.021–19.516). However, similar evidence was also reported in [8, 9]. Therefore, the variation might be the use of denominator for calculating latrine utilization rate in which the later studies use the total household including those that did not construct latrine, but it is appropriate to use households owned latrine as a denominator [7, 23]. In addition, a pooled result due to systematic reviews of different pocket studies was a reason for the low utilization rate. However, slightly lower evidence was reported from Hullet Ejj Enese of the Amhara region of Northern Ethiopia (92%) and Nepal (94.3%) [17, 29]. The discrepancy among the study findings might be related to climatic zones which are believed to affect the construction and durability of latrines for sustainable use [30–32]. Progressive sanitation facilities and good community awareness might be the reason in Nepal [29]. Similar reports were documented from Jimma of South West Ethiopia (87.8%), Hawassa town of Southern Ethiopia (85.4%), Wondogent of Southern Ethiopia (83%) [33, 34].

As evidenced in Kenya [35] and Ethiopia [36], more than 80% of latrine constructed in rural communities was not used more than one year due to their demolishing characteristics. A similar challenge was reported that forcing households to construct latrine during mass advocacy and campaign results in poor quality latrine construction at the community level. Therefore, it is essential to consider enabling factors for households to own and utilize latrine sustainably. This study reported that constructing latrine using slab floor was two times more likely owned by households than wood and mud floor pit latrines and similarly, three times for privately owned houses and occupation with government employees (Table 2). This finding was consistent with evidence from Hawusen, Dabat, Bahirdar, and Nepal [17, 24, 25, 33].

The scientific evidence shows that household members were four times as likely to use a latrine if it was located inside, rather than outside, the family compound [21, 22]. In this study, more than half of the latrines constructed were located inside the household compound. This was the reason for the high latrine utilization rate evidenced at the community level. Having these realities, program implementers should acknowledge health promoters and households to own latrine inside the household compound. Women Development Armies (WDAs) at the community level reported two times more influence to utilize latrine than health extension workers (HEWs). The reason might be health extension workers were overloaded at the health facility level for health care service provision resulting in poor support for the community than women development armies (WDAs) at the community level. Similar evidence was reported from other studies [33, 36, 37].

## 5. Conclusion

The study showed that households owned latrine at the community level were low. However, the majority of constructed latrines were utilized by household members. Adults and children were more adhered to utilize latrine. The desired level of latrine ownership and utilization would be realized if and only if all sanitation and hygiene components in the country's health extension package were kept on eye side by side in line with identified predictor factors.

## Figures and Tables

**Table 1:** Sociodemographics of households in rural villages of Tigray, Northern Ethiopia, 2018 (*n* = 756).

Characteristics	Frequency (%)
*Respondent role*	
Mother	527 (69.7)
Father	137 (18.1)
Adult daughter	91 (12.1)
*Gender*	
Male	182 (24.1)
Female	574 (75.8)
*Religious background*	
Orthodox	741 (98)
Muslim	11 (1.5)
Protestant	3 (0.4)
*Age category*	
<30 years	257 (44)
30–45 years	289 (38.2)
>45 years	191 (25.3)
*Educational status*	
Able to read and write	440 (58.2)
Unable to read and write	316 (41.7)
*Occupational status*	
Daily laborer	5 (0.7)
Farmer	676 (89.4)
Government employee	18 (2.4)
Merchant	21 (2.8)
Unemployed	36 (4.7)
*Marital status*	
Married	550 (72.8)
Single	93 (12.3)
Divorced	62 (8.2)
Widowed	48 (6.3)

**Table 2:** Latrine ownership and its characteristics among households in rural villages of Tigray, Northern Ethiopia, 2018 (*n* = 756).

Characteristics	Frequency (%)
*Household owned latrine*	
Yes	270 (35.7)
No	486 (64.3)
*Latrine type*	
Pit latrine with wood and mud floor	174 (64)
Pit latrine with slab floor	94 (34.8)
Ventilated improved pit latrine	2 (0.7)
*Latrine location*	
Inside the compound	177 (65.6)
Outside the compound	93 (34.4)
*Latrine distance from the house compound*	
<50 meters	245 (90.7)
≥50 meters	25 (9.3)
*Latrine utilization*	
Yes	228 (84.4)
No	42 (15.6)
*Household members utilized latrine*	
Only adults	33 (12.2)
Only children	2 (0.7)
Both adults and children	235 (87.1)
*Hand washing practice after latrine use*	
Yes	15 (5.6)
No	255 (94.4)
*Latrine with hand washing facility*	
Yes	40 (14.8)
No	230 (85.2)
*Households knew latrine importance*	
Yes	670 (88.6)
No	86 (11.4)
*Reasons for not constructing latrine*	
Lack of money	51 (6.7)
Lack of space	36 (4.8)
Demolished latrines	520 (68.8)
Lack of awareness	53 (7)
Others	96 (12.7)
*Households having information on latrine*	
Yes	549 (72.6)
No	207 (27.4)
*Source of information*	
Health extension workers	277 (50.5)
Radio	130 (23.7)
Women development armies	116 (21.1)
Others	26 (4.7)
*Possess radio*	
Yes	470 (62.2)
No	286 (37.8)

**Table 3:** Determinants of latrine ownership among households in rural villages of Tigray, Northern Ethiopia, 2018 (*N* = 756).

Variables	Latrine ownership			
	Latrine prevalence		Latrine utilization	
	COR (95% CI)	AOR (95% CI)	COR (95% CI)	AOR (95% CI)
*Occupation*				
Farmer	4.67 (1.072–20.423)^*∗*^*⊃*	4.27 (0.92–19.4)		
Government employee	8.50 ,(1.50448.049)^*∗*^	**3.542 (0.586–21.397)** ^*∗*^	0.27 (0.062–1.187)^*∗*^	0.25 (0.05–1.13)
Unemployed	1	1	1	1
*Marital status*				
Married	0.39 (0.154–0.995)^*∗*^	0.36 (0.13–0.95)		
Single	5 (0.872–28.663)	4.57 (0.75–27.22)		
Divorced	2 (0.117–34.096)	1.83 (0.099–32.39		
Widowed	1	1		
*Possess radio*				
Yes	0.82 (0.606–1.117)^*∗*^	0.75 (0.52–1.06)		
No	1	1		
*Latrine type*				
Slab floor pit			2.51 (0.103–4.512)^*∗*^	**1.790 (0.296–3.102)** ^*∗*^
Ventilated pit			1	1
*House ownership*				
Relative	3.05 (1.107–8.396)^*∗*^	2.79 (0.95–7.976)		
Private	3.09 (1.588–5.999)^*∗*^	**3.13 (1.528–6.401)** ^*∗*^		
Rental	1	1		
*Latrine location*				
Inside the household compound			3.991 (0.876–18.183)^*∗*^	**4.463 (1.021–19.516)** ^*∗*^
Outside the household compound			1	1
*Information source*				
Health extension workers (HEWs)	0.586 (0.389–0.884)^*∗*^	**1.902 (1.269–2.852)** ^*∗*^		
Women development armies (WDAs)	1.633 (0.422–2.952)^*∗*^	1.49 (0.36–2.80)	1.706 (1.1.29–2.579)^*∗*^	**2.425 (0.728–8.083)** ^*∗*^
Others	1	1	1	1

*Note*. Abbreviations: AOR: adjusted odds ratio, COR: crude odds ratio, *P*-value: precision value; statistical decisions: ^*∗*^ = *P*-value ≤0.05; ^*∗*^ = *P*-value <0.01, ^*∗*^ = *P*-value ≤0.001.

## Data Availability

The data are available upon reasonable request from the corresponding author.

## Abbreviations

EDHS: Ethiopian demographic health survey
AOR: Adjusted odds ratio
COR: Crude odds ratio
IEC: Information, education, and communication.
